# Correlation analysis between cerebral microangiopathy and autonomic nervous dysfunction

**DOI:** 10.1002/brb3.3391

**Published:** 2024-02-10

**Authors:** Na Liu, Hongmin Wang, Bing Han, Wenyuan Wang, Moqing Zhou, Lin Yang, Yanyong Wang

**Affiliations:** ^1^ Department of Neurology The First Hospital of Hebei Medical University Shijiazhuang Hebei China

**Keywords:** autonomic dysfunction, cerebral microangiopathy, correlation

## Abstract

**Objective:**

Our study was conducted aimed at investigating the potential correlation between cerebral microangiopathy and autonomic nervous dysfunction.

**Methods:**

We initially included 164 hospitalized patients with cerebral microangiopathy at our hospital from November 2019 to January 2021. Based on the inclusion and exclusion criteria, a final total of 162 patients with cerebral microangiopathy were selected. According to the patient's Autonomic Symptom Profile (ASP) score, patients with a score greater than 22 were categorized into a group with concomitant autonomic dysfunction (71 cases, combined group), while those with a score below 22 were categorized into a group of isolated cerebral microangiopathy (83 cases, cerebral microangiopathy group). The general data and laboratory examination results of the two groups were analyzed, and Pearson correlation analysis was performed to evaluate the correlation between cerebral microangiopathy and autonomic dysfunction, as well as the influencing factors of cerebral microangiopathy patients combined with autonomic dysfunction.

**Results:**

There were no significant differences between the two groups in terms of sex, BMI, smoking, drinking, family dementia history, diabetes, hypothyroidism, carotid atherosclerosis, obstructive sleep apnea hypopnea syndrome, hyperuricemia, hyperlipidemia, chronic obstructive pulmonary disease, Hamilton Anxiety Scale score, Hamilton Depression Scale score, 24‐h mean systolic blood pressure (SBP), 24‐h mean diastolic blood pressure DBP, daytime mean systolic blood pressure (dSBP), daytime mean diastolic blood pressure, nighttime mean systolic blood pressure (nSBP), nighttime mean diastolic blood pressure, 24‐h systolic blood pressure standard deviation (SBPSD), 24‐h diastolic blood pressure standard deviation, daytime diastolic blood pressure standard deviation, nighttime diastolic blood pressure standard deviation (nDBPSD), nDBPSD (*p* > .05). However, significant differences were observed between the two groups regarding age, history of coronary heart disease, hypertension, leukoaraiosis, cognitive function, ASP score, SSR, 24‐h SBPSD, daytime systolic blood pressure standard deviation (dSBPSD), nighttime systolic blood pressure standard deviation (nSBPSD), standard deviation of RR interval (SDNN), root mean square value of successive RR interval difference (RMSSD), high‐frequency component (HF), and low‐frequency component (LF) (*p* < .05). Moreover, the levels of TG, TC, HDL‐C, and LDL‐C did not show significant differences between the two groups (*p* > .05), but there were significant differences in blood uric acid and homocysteine (Hcy) levels (*p* < .05). Age, history of leukoaraiosis, cognitive function assessment, blood uric acid, Hcy levels, 24‐h SBPSD, dSBPSD, and nSBPSD showed positive correlations with ASP scores and SSR in patients with cerebral microangiopathy (*p* < .001). In contrast, hypertension, SDNN, RMSSD, HF, and LF showed negative correlations with ASP scores and SSR (*p* < .001). Moreover, coronary heart disease was negatively correlated with ASP scores but positively correlated with SSR (*p* < .001). The independent variables included age, history of leukoaraiosis, cognitive function assessment, ASP score, SSR, blood uric acid, Hcy, bradykinin, coronary heart disease, hypertension, 24‐h SBPSD, dSBPSD, nSBPSD, SDNN, RMSSD, HF, and LF, which were indicators with differences in general data and laboratory indicators. The dependent variable was patients with cerebral microangiopathy combined with autonomic nervous dysfunction. The analysis results showed that age, history of leukoaraiosis, ASP score, SSR, 24‐h SBPSD, dSBPSD, nSBPSD, SDNN, RMSSD, HF, and LF were the influencing factors of patients with cerebral microangiopathy complicated with autonomic nervous dysfunction.

**Conclusion:**

We demonstrates that age, history of leukoaraiosis, cognitive function assessment, blood uric acid, Hcy level, 24‐h SBPSD, dSBPSD, nSBPSD, blood pressure, SDNN, RMSSD, HF, LF, and coronary heart disease were highly associated with cerebral microangiopathy with autonomic dysfunction. Furthermore, the influencing factors of cerebral microangiopathy with autonomic dysfunction are age, history of leukoaraiosis, ASP score, SSR, blood pressure variability, and HRV.

## Graphical abstract

Patients with severe checkpoint inhibitor‐associated pneumonia (CIP) have a poor prognosis, and the main cause of death is tumor progression. Suspension of anti‐tumor therapy is the independent risk factors for poor prognosis of patients with CIP.

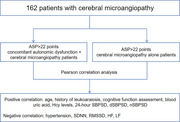

## 1 INTRODUCTION

Cerebral microangiopathy encompasses a group of conditions caused by small vascular abnormalities within the brain, such as small vessels, arterioles, small perforating arteries, and capillaries. It accounts for approximately 25% of ischemic cerebral microangiopathies and affects about 20% of the elderly population, significantly impacting human health and quality of life (Litak et al., 2020; Zanon Zotin et al., 2021). Typical imaging markers of cerebral microangiopathy include new subcortical lacunar infarction, cerebral microhemorrhages, perivascular space expansion, white matter lesions, and cerebral atrophy. Although some patients with cerebral microangiopathy may not exhibit obvious symptoms, others may experience acute clinical manifestations such as lacunar cerebral infarction, cerebral hemorrhage, or damage to various regions of the brain, resulting in cognitive disorders, emotional disturbances, gait abnormalities, and urinary incontinence (Kumar et al., 2022). Despite extensive research, the exact pathogenesis of cerebral microangiopathy remains incompletely understood. Age, hypertension, atherosclerosis, cerebral amyloid vascular disease, genetic disorders, and other factors are recognized as risk factors (Elyas et al., 2021).

The autonomic nervous system, which innervates all organs of the human body, plays a crucial role in regulating the body's response to various stressors, such as hypertension, hyperglycemia, acute ischemic stroke, and cerebral microangiopathy. It initiates the stress response, neutralizes the impact of stressors, and restores balance (Gibbons, 2019). However, when the demand for stability surpasses its adaptive capacity, imbalances between the sympathetic and parasympathetic branches of the autonomic nervous system can lead to dysfunctional responses or delayed responses, eventually resulting in stress‐related diseases, such as arrhythmias, myocardial infarction, and other complications (Goldberger et al., 2019). These stress‐related diseases, acting as secondary stressors, further disrupt the balance of the sympathetic and parasympathetic nerves, progressively increasing the risk of cardio‐cerebral microangiopathies. Previous studies have suggested that cerebral microangiopathy and autonomic nervous dysfunction form a detrimental closed‐loop cycle (Benarroch, 2020). As a stressor, cerebral microangiopathy continually impairs the function of the autonomic nervous system. In turn, the imbalanced autonomic nervous system promotes the occurrence and progression of cerebral microangiopathy, thereby increasing the overall burden (Baker et al., 2019). However, clinical studies exploring the relationship between cerebral microangiopathy and autonomic nervous dysfunction are scarce. Hence, the current study aimed to investigate the potential correlation between cerebral microangiopathy and autonomic nervous dysfunction.

## 2 MATERIALS AND METHODS

### 2.1 Clinical background

Analysis was conducted on 164 patients with cerebral microangiopathy at our hospital from November 2019 to January 2021. Based on the inclusion and exclusion criteria, a total of 162 patients with cerebral microangiopathy were ultimately selected as the research subjects. These patients were further divided into two groups based on their Autonomic Symptom Profile (ASP) (Expert Consensus Group on the Diagnosis & Treatment of Cerebral Small Vessel Disease, 2013) scores, using a cutoff of 22 points. Patients with a score greater than 22 were categorized into a group with concomitant autonomic dysfunction (71 cases, combined group), whereas those with a score below 22 were categorized into a group of isolated cerebral microangiopathy (83 cases, cerebral microangiopathy group). In the combined group, there were 31 males and 40 females, whereas in the cerebral microangiopathy group, there were 37 males and 46 females. The gender distribution between the two groups was comparable (*p* > .05). The research protocol was conducted in accordance with the relevant requirements of the Declaration of Helsinki by the World Medical Association. Prior to participation in the study, patients and their guardians provided informed consent, demonstrating their willingness to be included in the research.

### 2.2 Selection criteria

#### 2.2.1 Inclusion criteria

The inclusion criteria followed the diagnostic criteria for cerebral microangiopathies outlined in the Expert Consensus on the Diagnosis and Treatment of Cerebral Small Vessel Disease (Khaw et al., 2021). Patients with symptoms of cerebral microangiopathies who were diagnosed using CT scans were included. Symptoms included dizziness, fatigue, language disorders, disorders of consciousness, decreased memory, cognitive ability, emotional instability, and intellectual decline. A score of ASP greater than 22 indicated the presence of autonomic dysfunction. The age range for inclusion was between 45 and 80 years old.

#### 2.2.2 Exclusion criteria

The study excluded patients with hematological diseases, cerebral white matter abnormalities caused by hypoglycemia, vitamin B12 deficiency, atrophic gastritis, malignant tumors, severe liver or kidney dysfunction (defined as cardiac function grade IV and/or liver failure and/or kidney function uremia stage) related to diabetes, a history of stroke, carbon monoxide poisoning, hypoxic encephalopathy, brain or other organ tumors, multiple sclerosis, severe head injury, metabolic infectious encephalopathy, neurodegenerative diseases such as Alzheimer's disease and Parkinson's disease, aphasia, hearing or visual impairment, mental illness, severe alcohol addiction, and individuals using special drugs.

## 3 METHODS

(1) Clinical data of the patients were collected, including sex, age, BMI, drinking, smoking, family history of dementia, diabetes, coronary heart disease, hypertension, hypothyroidism, carotid atherosclerosis, obstructive sleep apnea hypopnea syndrome, hyperuricemia, hyperlipidemia, chronic obstructive pulmonary disease, history of leukoaraiosis, cognitive function assessment, ASP score, and SSR. (1) Cognitive function assessment (Baker et al., 2019): On the day of admission, a MoCA score examination was conducted to assess various cognitive domains, including attention and concentration, executive functions, memory, language, visuospatial abilities, abstract thinking, calculation, and orientation. A higher total score out of 30 indicates better cognitive function in the patient. (2) ASP score (Lu et al., 2018): The ASP score was established by Suarez. The participants filled out the scale themselves. In cases where independent completion was not possible, the experimenter assisted in filling out the scale by asking questions, with the experimenter calculating the score. An ASP score of ≤22 is considered within the normal range, whereas an ASP score of >22 indicates abnormal autonomic nervous system function. (3) SSR detection method (Tong et al., 2021): All subjects were instructed to abstain from medications that affect autonomic nervous function for 48 h prior to the examination. Additionally, consumption of coffee, strong tea, smoking, and strenuous exercise were prohibited for 4 h before the examination. Fasting for 2 h prior to the examination was also required. The examination took place in the electromyography room. The patient remained awake and relaxed, with the skin temperature controlled at 32–36°C using a skin thermometer (KFT61, Yueqing Rehabilitation Medical Equipment Factory). In cases of low skin temperature, a heated water bag was used to maintain warmth. The examination was performed using an electromyography machine (MEB‐9200K, Japanese Photoelectric), employing electrical stimulation with a stimulation duration of 0.2 ms, band‐pass range of 0.1–100 Hz, analysis time of 10 s, and sensitivity of 0.5–5.0 mV/cm (reading sensitivity of 0.5 mV/cm). The subject was positioned supine, with the palms of the hands and feet degreased using alcohol. If necessary, the local skin resistance was reduced by gently rubbing with fine sandpaper. Disc electrodes (provided with the electromyography machine) were applied with conductive paste. A recording electrode was placed on the palm, whereas a corresponding reference electrode was placed on the opposite palm, ensuring close contact with the skin. Adhesive tape was used to secure the electrodes, and a ground wire was placed approximately 10 cm away from the stimulation point. The stimulation current was adjusted to 20 mA, and the left wrist median nerve and the left ankle posterior tibial nerve were stimulated separately. Each part was stimulated twice with an interval of approximately 1 min. If no waveform was present, the interval should be at least 1 min, and the current should be gradually increased to 30 and 40 mA for stimulation. If a waveform was still not observed, it should be recorded as a missing waveform. The recorded graphics were automatically saved, and a waveform with a clear start and a high amplitude was selected to calculate the initial latency (time from stimulus to waveform start), amplitude (peak‐to‐valley value), and area (enclosed by the curve with the baseline as the reference line). In the measured graph, if the waveform latency exceeded ($\bar{x}$ + 2s), the amplitude was lower than ($\bar{x}$ + 2s), or the waveform was missing, it was considered abnormal, whereas other cases were considered normal. (4) Hamilton Anxiety Scale (HAMA) and Hamilton Depression Scale (HAMD) scores (Wang et al., 2021; Xu et al., 2022): The psychological status of anxiety and depression in all patients was evaluated using the HAMA and the HAMD. HAMA consists of 14 items, with each item scored on a 0–4 point 5‐level scale. A score of 7–14 indicates mild anxiety, 15–21 indicates moderate anxiety, and >21 indicates severe anxiety. HAMD consists of 17 items, also scored on a 0–4 point 5‐level scale. A score of 7–17 indicates mild depression, 18–24 indicates moderate depression, and >24 indicates severe depression. (5) Ambulatory blood pressure monitoring and blood pressure variability (BPV) analysis: A noninvasive portable ambulatory blood pressure monitor (1 mmHg = 0.133 kPa) was utilized to record the 24‐h dynamic changes in blood pressure for all patients. The measurements were taken every 30 min during the day (6:00–22:00) and every 60 min during the night (22:00–6:00). The subjects were not restricted in their daily activities, and measurement values greater than 80% were considered qualified. The dynamic blood pressure indicators assessed include: 24‐h mean systolic blood pressure (24‐h SBP), 24‐h mean diastolic blood pressure (24‐h DBP), daytime mean systolic blood pressure (dSBP), daytime mean diastolic blood pressure (dDBP), nighttime mean systolic blood pressure (nSBP), and nighttime mean diastolic blood pressure (nDBP). Furthermore, the standard deviation of blood pressure during each aforementioned time period was used as a long‐term BPV indicator, including 24‐h systolic blood pressure standard deviation (24‐h SBPSD), 24‐h diastolic blood pressure standard deviation (24‐h DBPSD), daytime systolic blood pressure standard deviation (dSBPSD), daytime diastolic blood pressure standard deviation (dDBPSD), nighttime systolic blood pressure standard deviation (nSBPSD), and nighttime diastolic blood pressure standard deviation (nDBPSD). (6) Dynamic ECG monitoring and HRV analysis: A synchronous 12‐lead dynamic ECG scanner was used to record the 24‐h dynamic changes in heart rate for time‐domain and frequency‐domain HRV analysis. Time‐domain indicators included the standard deviation of RR intervals (SDNN) within 24 h and the root mean square value of successive RR interval differences (RMSSD) within 24 h. Frequency‐domain indicators included the high‐frequency component (HF) and low‐frequency component (LF) of HRV.

(2) The follow‐up laboratory examination results of patients were collected, including TG, TC, HDL‐C, LDL‐C, blood uric acid, and homocysteine (Hcy).

(3) Pearson correlation analysis was employed to evaluate the correlation between cerebral microangiopathy and autonomic nervous dysfunction.

(4) The factors influencing autonomic nervous dysfunction in patients with cerebral microangiopathy were determined.

### 3.1 Statistical analysis

Data processing was conducted using SPSS 21.0 statistical software. If the measurement data followed a normal distribution, it was presented as mean ± standard deviation ($\bar{x}$ ± s). Intergroup comparisons were performed using two independent samples *t*‐tests or one‐way ANOVA. On the other hand, if the data did not follow a normal distribution, it was represented as median (quartile) [M (P25, P75)] and intergroup comparisons were made using Mann–Whitney analysis or the Kruskal–Wallis test. Count data were expressed as the number of cases (percentage) [*n* (%)] and group comparisons were conducted using the chi‐squared test or Fisher's exact probability method. Pearson correlation analysis was used to assess the correlation between cerebral microangiopathy and autonomic nervous dysfunction, with statistical significance set at *p* < .05.

## 4 RESULTS

### 4.1 Comparison of general information between the two groups

There were no significant differences in gender, BMI, smoking, drinking, family history of dementia, diabetes, hypothyroidism, carotid atherosclerosis, obstructive sleep apnea hypopnea syndrome, hyperuricemia, hyperlipidemia, chronic obstructive pulmonary disease, HAMA score, HAMD score, 24‐h SBP, 24‐h DBP, dSBP, dDBP, nSBP, nDBP, 24‐h SBPSD, 24‐h DBPSD, dDBPSD, nDBPSD, and nDBPSD between the two groups (*p* > .05). However, significant differences were observed in terms of age, history of coronary heart disease, hypertension, leukoaraiosis, cognitive function assessment, ASP score, SSR, 24‐h SBPSD, dSBPSD, nSBPSD, SDNN, RMSSD, HF, and LF (*p* < .05) (Table 1).

**TABLE 1:** Comparison of general information between two groups.

Parameter	Combined group (*n* = 71)	Cerebral microangiopathy group (*n* = 83)	*t*/*χ* ^2^	*p*
Sex (male)(*n*)	31	37	.551	.458
Age (year)	64.29 ± 14.39	58.01 ± 14.92	21.982	<.001
BMI (kg/m^2^)	23.09 ± 3.02	23.21 ± 3.16	1.468	.225
Alcohol consumption (*n*)	18	22	.026	.872
Tobacco use (*n*)	15	20	.192	.661
Family dementia history (*n*)	7	6	.342	.558
Diabetes (*n*)	13	18	.271	.603
Coronary heart disease (*n*)	34	21	8.502	.004
Hypertension (*n*)	49	34	12.117	<.001
Hypothyroidism (*n*)	7	10	.187	.665
Carotid atherosclerosis (*n*)	25	28	.037	.847
OSAHA (*n*)	8	10	.023	.879
Hyperuricemia (*n*)	8	11	.139	.709
Hyperlipidemia (*n*)	9	13	.279	.597
COPD (*n*)	6	9	.249	.618
Leukoaraiosis (*n*)	51	38	10.644	.001
Cognitive function (point)	20.37 ± 2.45	24.09 ± 2.56	−16.391	<.001
ASP score	25.09 ± 2.87	14.39 ± 2.41	38.091	<.001
Abnormal SSR (*n*)	52	19	39.038	<.001
HAMA score (point)	5.93 ± 0.36	6.03 ± 0.41	−.871	.287
HAMD score (point)	5.87 ± 0.43	6.08 ± 0.48	−.937	.279
24‐h SBP (mmHg)	123.09 ± 19.08	125.77 ± 18.67	−.767	.371
24‐h DBP (mmHg)	71.82 ± 8.92	75.09 ± 9.04	−.387	.831
Dsbp (mmHg)	121.06 ± 17.23	128.33 ± 18.47	−.462	.069
dDBP (mmHg)	72.07 ± 10.38	76.84 ± 11.09	−.381	.076
nSBP (mmHg)	120.78 ± 15.93	120.11 ± 16.09	3.281	.096
nDBP (mmHg)	68.93 ± 9.09	69.09 ± 7.48	−.612	.381
24‐h SBPSD (mmHg)	14.76 ± 2.31	12.09 ± 2.27	27.651	<.001
24‐h DBPSD (mmHg)	10.73 ± 1.93	10.18 ± 2.65	.612	.357
dSBPSD (mmHg)	13.78 ± 2.46	12.03 ± 2.53	11.928	<.001
dDBPSD (mmHg)	10.05 ± 2.38	9.53 ± 2.39	.651	.362
nSBPSD (mmHg)	13.48 ± 3.02	10.73 ± 3.52	36.981	<.001
nDBPSD (mmHg)	9.25 ± 2.02	8.73 ± 2.17	.761	.276
SDNN (ms)	104.39 ± 17.92	148.93 ± 19.48	−26.287	<.001
RMSSD (ms)	23.76 ± 10.93	35.98 ± 13.29	−76.198	<.001
HF	105.82 ± 37.19	292.91 ± 39.81	−56.391	<.001
LF	172.93 ± 21.82	367.87 ± 34.19	−48.917	<.001

Abbreviations: ASP, autonomic symptom profile; dbp, diastolic blood pressure; dSBP; daytime mean systolic blood pressure; dDBP, daytime mean diastolic blood pressure; DBPSD, diastolic blood pressure standard deviation; dSBPSD, daytime systolic blood pressure standard deviation; dDBPSD, daytime diastolic blood pressure standard deviation; HAMA, Hamilton anxiety scale; HAMD, Hamilton depression scale; HF, high‐frequency component; LF, low‐frequency component; nDBP, nighttime mean diastolic blood pressure; nSBP, nighttime mean systolic blood pressure; nSBPSD, nighttime systolic blood pressure standard deviation; nDBPSD, nighttime diastolic blood pressure standard deviation; RMSSD, root mean square value of successive RR interval differences; SBPSD, systolic blood pressure standard deviation; sbp, systolic blood pressure; SDNN, standard deviation of RR intervals.

### 4.2 Comparison of laboratory examination results between two groups

There were no significant differences in the levels of TG, TC, HDL‐C, and LDL‐C (*p* > .05), whereas significant differences were found in blood uric acid and Hcy levels between the two groups (*p* < .05), as shown in Table 2.

**TABLE 2:** Comparison of laboratory examination results between two groups (±s).

Parameter	Combined group (*n* = 71)	Cerebral microangiopathy group (*n* = 83)	*t*	*p*
TG (mmol/L)	1.83 ± 0.47	1.77 ± 0.51	2.915	.088
TC (mmol/L)	4.29 ± 0.56	4.18 ± 0.59	12.021	<.001
HDL‐C (mmol/L)	1.07 ± 0.25	1.16 ± 0.31	−18.981	<.001
LDL‐C (mmol/L)	1.19 ± 0.31	1.25 ± 0.29	17.092	<.001
Blood uric acid (μmol/L)	218.91 ± 37.08	301.38 ± 39.76	−167.981	<.001
Hcy (μmol/L)	19.39 ± 3.29	15.03 ± 3.11	19.761	<.001

Abbreviation: Hcy, Homocysteine.

### 4.3 Correlation analysis of cerebral microangiopathy and autonomic nervous dysfunction

In patients with cerebral microangiopathy, age, history of leukoaraiosis, cognitive function, blood uric acid, Hcy levels, 24‐h SBPSD, dSBPSD, and nSBPSD showed positive correlations with ASP scores and SSR (*p* < .001). On the other hand, hypertension, SDNN, RMSSD, HF, and LF demonstrated negative correlations with ASP scores and SSR (*p* < .001). Coronary heart disease was negatively correlated with ASP scores, whereas it was positively correlated with SSR (*p* < .001) (Table 3).

**TABLE 3:** Correlation analysis between cerebral microangiopathy and autonomic nervous dysfunction.

Parameter	ASP score		SSR	
	*r* Value	*p* Value	*r* Value	*p* Value
Age	.518	<.001	.617	<.001
Coronary heart disease	−.058	.487	.017	.869
Hypertension	−2.187	<.001	−1.754	<.001
History of leukoaraiosis	.371	<.001	.276	<.001
Cognitive function	.822	<.001	.793	<.001
Blood uric acid	11.271	<.001	4.651	<.001
Hcy	7.632	<.001	7.038	<.001
Bradykinin	3.921	<.001	1.076	<.001
24‐h SBPSD	8.832	<.001	5.932	<.001
dSBPSD	6.321	<.001	4.392	<.001
nSBPSD	3.009	<.001	1.923	<.001
SDNN	−3.291	<.001	−2.761	<.001
RMSSD	−1.832	<.001	−0.981	<.001
HF	−1.652	<.001	−4.923	<.001
LF	−1.875	<.001	−7.472	<.001

Abbreviation: dSBPSD, daytime systolic blood pressure standard deviation; LF, low‐frequency component; RMSSD, root mean square value of successive RR interval differences; nSBPSD, nighttime systolic blood pressure standard deviation; SDNN, standard deviation of RR intervals.

### 4.4 Influencing factors of autonomic nervous dysfunction in patients with cerebral microangiopathy

The independent variables considered were age, history of leukoaraiosis, cognitive function, ASP score, SSR, blood uric acid, Hcy, bradykinin, coronary heart disease, hypertension, 24‐h SBPSD, dSBPSD, nSBPSD, SDNN, RMSSD, HF, and LF, which were selected based on differences observed in general data and laboratory indicators. The dependent variable was the presence of autonomic nervous dysfunction in patients with cerebral microangiopathy. The results indicated that age, history of leukoaraiosis, ASP score, SSR, 24‐h SBPSD, dSBPSD, nSBPSD, SDNN, RMSSD, HF, and LF were the influencing factors for cerebral microangiopathy patients with autonomic nervous dysfunction, as shown in Table 4.

**TABLE 4:** Influencing factors of autonomic nervous dysfunction in patients with cerebral microangiopathy.

Variable	β	SE	Waldx2	OR (95%CI)	*p* Value
Age	.823	.156	27.167	2.276 (1.445–3.013)	<.001
History of leukoaraiosis	.179	.353	22.079	4.865 (2.365–10.376)	<.001
Cognitive function	.089	.159	0.345	.913 (.659–1.332)	.571
ASP score	1.109	.248	21.298	2.398 (1.276–3.376)	<.001
SSR	.837	2.219	9.872	2.198 (1.531–2.876)	.001
Blood uric acid	.239	.169	12.182	1.287 (1.098–1.871)	.139
Hcy	.089	.159	0.345	.913 (.659–1.332)	.571
Bradykinin	.025	.216	0.017	1.029 (.678–1.479)	.916
Coronary heart disease	.629	1.121	0.329	1.883 (.281–3.368)	.573
Hypertension	.138	.109	1.851	1.138 (.922–1.379)	.171
24‐h SBPSD	.383	.172	2.169	1.452 (1.043–1.893)	.019
dSBPSD	.026	.001	2.253	1.023 (1.019–1.043)	<.001
nSBPSD	.889	.169	21.079	.533 (0.278–.898)	<.001
SDNN	−.779	.309	6.757	2.089 (1.209–3.869)	.006
RMSSD	−.262	.124	4.463	1.235 (.979–1.949)	.023
HF	−.624	.172	3.216	.523 (.371–.867)	.005
LF	−.619	.174	3.201	.524 (.373–.875)	.005

Abbreviations: ASP, Autonomic Symptom Profile; dSBPSD, daytime systolic blood pressure standard deviation; HF, high‐frequency component; Hcy, homocysteine; LF, low‐frequency component; nSBPSD, nighttime systolic blood pressure standard deviation.

## 5 DISCUSSION

Cerebral microangiopathy is an age‐related cerebrovascular disease that primarily affects arterioles, small veins, and capillaries within the brain. Imaging findings typically show high signal in the brain's white matter, lacunar infarctions, perivascular space enlargement, microhemorrhages, and cerebral atrophy (Jimenez‐Ruiz et al., 2021). The clinical manifestations of cerebral microangiopathy are diverse, ranging from acute stroke to cognitive impairment, emotional disturbances, sleep disturbances, gait abnormalities, and urinary dysfunction. These complications greatly impact daily functioning and reduce overall quality of life. Autonomic dysfunction is a common characteristic of neurodegenerative synucleinopathies (Jimenez‐Ruiz et al., 2021). Research has indicated a connection between autonomic nervous dysfunction and the pathological presence of alpha‐synuclein (De Maria et al., 2021). Neuropathological studies have identified Lewy bodies in significant locations such as the locus coeruleus, sympathetic ganglia, and parasympathetic nervous system plexus (Lai et al., 2020). Moreover, the autonomic nervous system, with its intricate anatomical and functional network within the brain and spinal cord, exerts significant influence on normal and abnormal cardiovascular functions through various pathways (Lai et al., 2020). Therefore, investigating the correlation between cerebral microangiopathy and autonomic nervous dysfunction is an important issue in clinical research.

Age serves as an independent predictor of autonomic dysfunction. As individuals age, autonomic control weakens, leading to an imbalance in sympathetic and parasympathetic nervous system activity in favor of the sympathetic side. These changes contribute to the decline in adaptability among the elderly and form the foundation for the development of cardiovascular diseases (Mercier et al., 2022). Autonomic nervous dysfunction is prevalent in various neurological disorders; however, it often receives limited attention in clinical practice due to its nonspecific and diverse symptoms and the absence of directly measurable objective indicators (Pfenniger et al., 2021). The autonomic nervous system has an extensive and profound anatomical structure. In clinical practice, objective values obtained by assessing changes in target organs during different tasks are often utilized to evaluate autonomic nervous function (Tamuli et al., 2021). Common approaches include the use of the ASP scoring scale, as well as tests assessing sympathetic and parasympathetic nervous system functions such as ambulatory blood pressure monitoring and skin sympathetic response assessments. BPV, also known as blood pressure volatility, is used to assess the extent of blood pressure fluctuations over a specific time period (Lu et al., 2018). Based on the duration of monitoring, BPV can be broadly categorized into short‐term BPV and long‐term BPV. Short‐term BPV involves the use of 24‐h ambulatory blood pressure monitoring to evaluate blood pressure fluctuations at different time intervals within a 24‐h period. Long‐term BPV monitoring typically extends beyond one week (Schneider & Schwerdtfeger, 2020). The relative stability of BPV reflects the dynamic equilibrium of sympathetic and parasympathetic nerve functions. Both BPV and HRV have gained significant attention as noninvasive examination methods in recent years and hold crucial value in assessing the effectiveness and prognosis of cardiovascular diseases, diabetes, metabolic syndrome, and other conditions (Schneider & Schwerdtfeger, 2020). BPV quantifies the degree of blood pressure fluctuation within a specific time period, typically expressed as the standard deviation of mean blood pressure over that period. On the other hand, HRV measures variations in the time interval between successive heartbeats, reflecting the modulation of cardiovascular autonomic function. Analysis methods for HRV primarily include time domain analysis and frequency domain analysis (Marchioni et al., 2018). Although previous clinical studies have predominantly focused on BPV and HRV in Parkinson's disease patients, limited research has been conducted on their association with cerebral microangiopathy. The white matter of the brain is particularly vulnerable to the impact of blood pressure fluctuations or inconsistent cerebral perfusion due to its relatively poor anastomotic system of perforating vessels originating from the cortex and pia mater arteries (Qiang et al., 2014). An epidemiological study has revealed that the risk of cerebral microangiopathy increases with elevated blood pressure levels and blood pressure fluctuations. Long‐term significant blood pressure fluctuations may result in an increased risk of leukoencephalopathy (Jie & Shan, 2017). Moreover, a longitudinal study conducted in the general population found that systolic blood pressure remained associated with the progression of white matter lesions even after considering the baseline white matter lesion load (Cortese et al., 2020). The results of this study demonstrated significant differences (*p* < .05) when comparing the age, presence of coronary heart disease, hypertension, history of leukoaraiosis, cognitive function, ASP score, SSR, 24‐h SBPSD, dSBPSD, nSBPSD, SDNN, RMSSD, HF, and LF between the two groups. These findings suggest that patients with cerebral microangiopathy complicated by autonomic nervous dysfunction may tend to be older and have a higher incidence of coronary heart disease, hypertension, and leukoaraiosis. Additionally, they exhibited lower cognitive function scores, higher ASP scores, higher abnormal rates of SSR, higher levels of 24‐h SBPSD, dSBPSD, nSBPSD, and lower levels of SDNN, RMSSD, HF, and LF.

Uric acid is a metabolic byproduct of purine metabolism (Bosevski et al., 2020). Several studies have highlighted that increased uric acid concentrations can induce oxidative stress in cerebrovascular endothelial cells, leading to white matter damage and cerebral microangiopathy (Bosevski et al., 2020). Hcy was found to promote atherosclerosis in the brain and contribute to cerebral microangiopathy (Yanan et al., 2019). Research conducted by Wang et al. showed that patients with cerebral microangiopathy exhibited elevated levels of uric acid, which were closely associated with vascular cognitive impairment (Huiqing et al., 2019). Another study reported that patients with cerebral microangiopathy had decreased blood uric acid levels and increased Hcy levels, which were greatly linked to cardiac autonomic neuropathy (Wenzheng et al., 2019). The findings of this study indicated significant differences in serum uric acid and Hcy levels between the two groups (*p* < .05), suggesting that patients with cerebral microangiopathy and autonomic dysfunction had lower uric acid levels and higher Hcy levels.

One study identified age as one of the influencing factors of autonomic dysfunction in patients (Yongzhi et al., 2022). Zhang et al. (Jiangqiong, 2017) highlighted that BPV and HRV served as indicators reflecting cardiovascular autonomic dysfunction in patients with Parkinson's disease, indicating a strong correlation between the two conditions. Another study emphasized the significant association between cerebral leukoaraiosis caused by cerebral microangiopathy and cognitive impairment, suggesting that assessing the condition of cerebral leukoaraiosis may facilitate early detection of cognitive impairment in patients (Cortese et al., 2020). The study by Ke et al. (Piętak & Rechberger, 2022) highlighted the close relationship between autonomic nervous dysfunction in patients with Parkinson's disease and SSR as well as HRV. Based on our study, age, history of leukoaraiosis, cognitive function, blood uric acid and Hcy levels, 24‐h SBPSD, dSBPSD, and nSBPSD were positively correlated with ASP scores and SSR in patients with cerebral microangiopathy. Conversely, factors such as hypertension, SDNN, RMSSD, HF, and LF, and coronary heart disease exhibited a negative correlation with ASP scores, whereas coronary heart disease showed a positive correlation with SSR (*p* < .001). These findings indicate that age, history of leukoaraiosis, cognitive function, blood uric acid, Hcy levels, 24‐h SBPSD, dSBPSD, nSBPSD, blood pressure, SDNN, RMSSD, HF, LF, and coronary heart disease are highly associated with cerebral microangiopathy accompanied by autonomic nervous dysfunction.

Piętak and Rechberger (2022) pointed out that HRV, SSR, and age are influencing factors in patients with autonomic nervous dysfunction. Sikorsky et al. (2019) suggested that HRV is a relevant factor in patients with autonomic dysfunction. Additionally, studies examining autonomic nervous dysfunction in patients with leukoencephalopathy during sleep depicted that sympathetic nervous system overactivity contributes to the development of leukoencephalopathy in OSA patients (Sikorsky et al., 2019). Their studies demonstrate an independent association between reduced nocturnal HRV and moderate to severe white matter lesions, indicating that nocturnal HRV can serve as a reliable indicator for detecting neuroimaging features in cerebral small vessel disease. They have provided evidence supporting the relationship between autonomic nervous dysfunction and the development of cerebral small vessel disease. Our results suggest that the influencing factors in patients with cerebral microangiopathy complicated by autonomic nervous dysfunction are age, history of leukoaraiosis, ASP score, SSR, 24‐h SBPSD, dSBPSD, nSBPSD, SDNN, RMSSD, HF, and LF. Additionally, age, history of leukoaraiosis, ASP score, SSR, BPV, and HRV are identified as potential influencing factors.

However, it is important to acknowledge the limitations of this study, such as the small sample size and its single‐center design. Future research should consider expanding the sample size and conducting multicenter randomized controlled trials. Furthermore, further investigation is needed to elucidate the underlying mechanisms linking age, history of leukoaraiosis, ASP score, SSR, BPV, and HRV in patients with cerebral microangiopathy combined with autonomic nervous dysfunction.

To conclude, patients with cerebral microangiopathy complicated by autonomic nervous dysfunction tend to be older and have a higher prevalence of coronary heart disease, hypertension, leukoaraiosis, lower cognitive function scores, higher ASP scores, increased abnormal SSR rates, elevated levels of 24‐h SBPSD, dSBPSD, nSBPSD, and decreased levels of SDNN, RMSSD, HF, and LF. Additionally, they exhibit lower uric acid levels and higher Hcy levels. Age, history of leukoaraiosis, cognitive function, blood uric acid, Hcy levels, 24‐h SBPSD, dSBPSD, nSBPSD, blood pressure, SDNN, RMSSD, HF, LF, and coronary heart disease exert significant association with cerebral microangiopathy accompanied by autonomic dysfunction. The influencing factors of cerebral microangiopathy with autonomic dysfunction include age, history of leukoaraiosis, ASP score, SSR, BPV, and HRV.

## AUTHOR CONTRIBUTIONS

Yanyong Wang contributed to the conception and design of the study. Na Liu and Hongmin Wang organized the database and organized the database. Bing Han, Wenyuan Wang, Moqing Zhou, and Lin Yang interpreted the data and searched the literature. Na Liu and Hongmin Wang wrote the first draft of the manuscript. Yanyong Wang edited the manuscript. All authors reviewed and approved the final version of the manuscript.

## CONFLICT OF INTEREST STATEMENT

The authors declare no conflicts interest.

### PEER REVIEW

The peer review history for this article is available at https://publons.com/publon/10.1002/brb3.3391

## Data Availability

The data and materials used and/or analyzed during the current study are available from the corresponding author on reasonable request.
